# Assessment of Prosodic Communicative Efficiency in Parkinson's Disease As Judged by Professional Listeners

**DOI:** 10.4061/2011/129310

**Published:** 2011-09-28

**Authors:** Heidi Martens, Gwen Van Nuffelen, Patrick Cras, Barbara Pickut, Miet De Letter, Marc De Bodt

**Affiliations:** ^1^Rehabilitation Centre for Communication Disorders, Antwerp University Hospital, Wilrijkstraat 10, 2650 Edegem, Belgium; ^2^Department of Neurology, Antwerp University Hospital, Wilrijkstraat 10, 2650 Edegem, Belgium; ^3^Centre for Speech and Hearing Disorders, Ghent University Hospital, De Pintelaan 185, 9000 Ghent, Belgium; ^4^Department of Speech and Language Pathology and Audiology, Ghent University, De Pintelaan 185, 9000 Ghent, Belgium

## Abstract

This study examines the impact of Parkinson's disease (PD) on communicative efficiency conveyed through prosody. A new assessment method for evaluating productive prosodic skills in Dutch speaking dysarthric patients was devised and tested on 36 individuals (18 controls, 18 PD patients). Three professional listeners judged the intended meanings in four communicative functions of Dutch prosody: Boundary Marking, Focus, Sentence Typing, and Emotional Prosody. Each function was tested through reading and imitation. Interrater agreement was calculated. Results indicated that healthy speakers, compared to PD patients, performed significantly better on imitation of Boundary Marking, Focus, and Sentence Typing. PD patients with a moderate or severe dysarthria performed significantly worse on imitation of Focus than on reading of Focus. No significant differences were found for Emotional Prosody. Judges agreed well on all tasks except Emotional Prosody. Future research will focus on elaborating the assessment and on developing a therapy programme paralleling the assessment.

## 1. Introduction

Dysarthria is a frequent complication of Parkinson's disease (PD). PD has been reported to affect speech in at least 60% of PD patients, with increased prevalence as the disease progresses [1]. Parkinsonian speech is mainly characterised by the impairment of voice, articulation, and prosody [1, 2]. This multidimensional impairment can have a negative impact on speech intelligibility and hence on communication and on quality of life.

Previous research points out that, next to articulation, prosody is the second most important factor contributing to speech intelligibility in dysarthric patients in general [3]. Prosody commonly refers to the suprasegmental speech signal aspects used to convey meaning through variation in fundamental frequency (F0), intensity, and duration [4–7]. In dysarthric PD patients specifically, six out of ten most deviant speech dimensions are associated with prosody: monopitch, reduced stress, monoloudness, inappropriate silences, short rushes of speech, and variable rate [1]. Therefore, assessing prosodic skills is important in the diagnosis and remediation of speech disorders in PD.

Nevertheless, scientific interest in the phenomenon of prosody within the field of speech-language pathology is rather scarce and slowly evolving, when it comes to characterisation, assessment, and intervention of atypical prosody [8–10]. During the last decade, the majority of publications focussing on prosody in PD evaluated prosody in terms of its formal dimensions, such as aspects of F0, intensity, and/or duration [2, 6, 11–14], and lately also voice quality and degree of reduction [15].

Nowadays, however, a more functional approach of prosody is advocated, emphasizing the importance of assessing prosodic communicative efficiency [8, 16]. For example, in the case of focus, a functional assessment will tell us whether or not a patient is able to highlight the most important information in an utterance and get this meaning across to listeners, abstracting away from the specific constellation of F0, intensity, and duration parameters being used.

Functional tasks for assessing prosody have already been described before, but few attempts have been made so far to bundle them into a standardised, comprehensive test battery. Peppé and McCann [17] faced the challenge and developed “Profiling Elements of Prosodic Systems-Children” (PEPS-C), a standardised instrument for assessing prosodic deficits in children. Four communicative functions conveyed by English prosody are tested: turn-end type (question-statement contrast), affect (liking-disliking contrast), chunking (marking syntactic boundaries), and focus (contrastive stress) [7, 17, 18].

The number of studies regarding communicative functions of prosody in PD is limited. An interesting study by Pell and colleagues [5] investigated the impact of PD on productive prosody. Naïve listeners judged the intended meanings conveyed by prosody in PD patients and healthy speakers in four speech contexts: phonemic stress, contrastive stress, sentence mode, and emotional prosody. Findings indicated that listeners consistently experienced greater trouble in recognising the intended meanings produced by PD speakers compared to healthy speakers.

Two other studies evaluated a specific aspect of prosodic meaning. Schröder and colleagues [19] investigated the perception of emotional prosody in PD and concluded that PD patients, compared to healthy individuals, performed significantly worse in classifying utterances according to their emotional prosody. Ma and colleagues [20] studied the production of the question-statement contrast through intonation in a group of PD speakers. They found that the question mode, compared to the statement mode, got across to listeners significantly worse.

The purpose of the current study is twofold: firstly, to present a method to assess the production of prosody in Dutch speaking dysarthric patients and, secondly, to gather further insight in the production of prosody in PD patients specifically.

In developing the assessment procedure, three general design principles were decided on. A first major decision concerned the choice for a functional approach of prosody, in line with Peppé [8] and Turk [16]. We believe that such an approach is very valuable for use in clinical practice and directly relevant to speech intelligibility and communication, because it has the potential of telling us whether or not a patient is able to convey meaning through prosody, regardless of the used prosodic forms. It allows for the possibility of “cue trading” or varying the relative salience of acoustic prosodic cues [4, 21]. This is what speech disordered patients do when they are trying to compensate for cues over which they have little or no control. A monotone PD speaker might, for example, still be able to convey contrastive stress successfully, not so much by marking the stressed word with a pitch movement (F0 cue) but mainly by lengthening it (duration cue).

This phenomenon has already been observed in dysarthric individuals with cerebral palsy (CP). CP speakers with severe dysarthria demonstrated a preserved ability to mark the question-statement contrast in short utterances, despite reduced control over F0, by producing the last syllable in a louder and longer manner than healthy speakers did [21, 22]. CP speakers also successfully conveyed contrastive stress to unfamiliar listeners, despite reduced F0 and intensity variation, by relying more heavily on duration [4].

A second decision pertained to the selection of prosodic functions. The functions of prosody in Dutch are generally fairly akin to those in English.  Table 1 provides an overview of Dutch prosodic functions [23] and their perceptual correlates. The lexical function of prosody in Dutch is somewhat less important than in English, since Dutch has considerably less compounds and phrases which need discriminating by means of stress, as in “a wetsuit” versus “a wet suit.” We adopted all functions listed in Table 1 in our own assessment, with the exception of one function, intentional marking, which we feared might be too difficult to instruct succinctly and unambiguously during testing.

A final decision concerned the choice of elicitation method. It can be argued that spontaneous speech is most sensitive to prosodic abnormalities [24], but spontaneous speech samples make it practically impossible to judge in a reliable and standardised manner to what extent the appropriate use of prosody could on its own disambiguate the meaning of an utterance [17].

Earlier research on reading as an elicitation method has shown that read speech prosody and spontaneous speech prosody are different [24–26], although very recently Ma and colleagues [15] found no significant differences between reading (sentences, passages) and monologue when perceptually rating dysprosody in PD patients. Previous publications on prosody assessment [5, 17, 27] mention the use of pictorial stimuli, which make the procedure usable for young children or other nonreading individuals. Our choice for written stimuli was motivated from a clinical point of view. First of all, reading is a commonly used speech therapy method, especially with adult patients, and, second, written stimuli allow for a quicker and easier development of a therapy programme mirroring the assessment test items.

A recent review of the research literature concerning treatment of prosody [10] lists imitation among the four most common treatment techniques. Nevertheless, very little systematic information is available in the literature about imitation as a method for evaluating prosodic abilities, compared to other types of stimuli. Möbes et al. [13] evaluated emotional speech in PD speakers, who produced the name “Anna” in a neutral, happy, or sad manner as requested by a visual cue on a monitor or alternatively imitated a professional speaker saying the name in the aforementioned ways. Patients performed worse than control speakers on the production task, but nearly normal on the imitation task. Peppé and McCann [17] explicitly opt for imitation tasks, next to functional tasks, allowing the clinician to discover whether a person has a certain prosodic form in his repertoire. We consider this a useful diagnostic procedure, because it enables the clinician to localise the prosodic problem: a patient can have trouble with the prosodic form (e.g., is he able to realise a final rise at the end of a question?), the meaningful application of prosodic form when expressing prosodic functions (e.g., can the patient make a clear question-statement contrast?), or both.

The present study aimed to address the following research questions.

1. Do healthy and PD speakers perform differently on expressing prosodic functions?
2. Does the elicitation method type (reading versus imitation) have any effect on prosodic performances: (a) between speaker groups and (b) within speaker groups?

## 2. Materials and Methods

### 2.1. Speech Elicitation Tasks

Taking into account the particulars of prosodic functions in Dutch, we designed a test battery assessing five important communicative functions of Dutch prosody: lexical stress, boundary marking, focus, sentence typing, and emotional prosody. The function of lexical stress has not been analysed yet, but Table 2 offers an overview of the other four functions which can already be reported on in this study.

The tasks used for elicitation of speech include reading tasks as well as imitation tasks. Both elicitation methods make use of exactly the same stimuli, which are fully listed in Table 2. The English example sentences given below and in Table 2 are translations of the Dutch test items.

The *Boundary Marking* function refers to the ability of a speaker to indicate syntactic boundaries through prosody. This ability was tested in three sentence pairs. Pair 1 contrasts a sentence containing a single object (e.g., “He bought a coat”) with a sentence containing a list of three objects (e.g., “He bought a coat, trousers, and a sweater”). Pairs 2 and 3 contrast sentences in which a phrase or subclause comes either at the beginning or at the end of the sentence (“No news is good news” versus “At this moment there is no news” and “When I'm ill, I stay at home” versus “I stay at home, when I'm ill”).

The *Focus* function has to do with the ability of a speaker to highlight the most important information of an utterance through prosody. The speaker is presented with three pairs of sentences in which one (typographically marked) keyword needs to be emphasised. In each sentence pair a different word has to be stressed (e.g., “Maybe Pete's on holiday” versus “Maybe Pete's on holiday”).

The *Sentence Typing* function deals with the ability of a speaker to use prosody in order to distinguish between a statement and a question. Three short utterances were selected that had to be produced as a declarative sentence (e.g., “Karen plays tennis”) and also as a declarative question (e.g., “Karen plays tennis?”).

Finally, the *Emotional Prosody* function refers to the ability of a speaker to express emotion through prosody. To this end the speaker has to produce a target utterance (e.g., “It's almost time”) in four different manners: neutral, angry, happy, and sad. In order to facilitate the production of emotion on command, the target utterance is preceded by an appropriate introductory phrase (angry: “Hurry up!”; happy: “Fantastic!”; and sad: “Too bad”).

### 2.2. Speakers

Speech samples were elicited from 36 Dutch-speaking adults: 18 individuals diagnosed with Parkinson's disease (PD) and 18 healthy control subjects (CS). PD participants were recruited through hospitals, patient organisations, and speech-language pathologists in private practice, and participated on a voluntary basis. The PD group included 7 men and 11 women (age range 47–82; average age 63; median age 63). The CS group was matched for gender and equally consisted of 7 men and 11 women (age range 36–75; average age 60; median age 63).  Table 3 lists the available PD speaker details. Unfortunately, further information on the patients' medical history and current medical status could not systematically be obtained, considering the various ways in which patients had been recruited.

The first author rated the global severity of the dysarthria of PD speakers on a four-point grading scale (0 = normal; 1 = mild; 2 = moderate; 3 = severe), before they actually took the prosody test, on the basis of an elicited conversation. The majority of PD speakers demonstrated a mild dysarthria, one third presented with a moderate form of dysarthria and in one subject a severe dysarthria was observed.

All participants were native speakers of Dutch, speaking different Flemish regional accents. They all reported sufficient visual skills in order to take the test, with the exception of one PD participant who was not able to fulfil the reading task in a reliable manner due to sight problems and was consequently only tested by means of the imitation task. Cognitive skills were not explicitly screened, but all participants demonstrated sufficient abilities to understand and execute the instructions of the assessment. Hearing skills were not explicitly screened either, but all participants reported sufficient abilities to understand the oral instructions and to hear the prerecorded model utterances of the imitation task.

Samples were recorded in a quiet environment, with an AKG (C555L) headset microphone connected to a Dell Vostro laptop computer, an external sound card (E-MU 0404 USB) and Audacity software (freely available, sampling rate 44.1 kHz, 24 bit, mono). Afterwards, each test item was saved as a separate wav-file using Audacity software.

All speakers took the test in the same fixed order: all of them were first asked to undertake the reading task and then the imitation task. For the reading task, subjects were instructed to read aloud stimulus words and sentences on a separate sheet of paper. No special instructions were given for the Boundary Marking items. For the Focus test items, participants were instructed to stress the typographically highlighted word. For the Sentence Typing items, speakers were instructed to make the declarative questions sound like genuine questions. For the Emotional Prosody task, an instruction concerning the specific target emotion preceded every item.

For the imitation task, subjects listened to stimulus words and sentences identical to the ones in the reading task, prerecorded by a professional speaker, and presented through the Dell Vostro computer speakers. Participants were instructed to imitate the utterances, not only by repeating them literally but also by adopting the way in which they were said.

### 2.3. Preparation of the Speech Material for Perceptual Evaluation

The Sentence Typing and Focus tasks consist of prosodic minimal pairs (see Table 2). Parts of the Boundary Marking samples were removed for perceptual evaluation, in order to obtain prosodic minimal pairs for this function as well (removed parts are between square brackets in Table 2). Prosodic boundaries can be realised with a pause, a boundary marking pitch movement, or both. Additionally, there is always a certain amount of pre-boundary lengthening [25]. The sample parts selected for perceptual evaluation consisted of utterances containing a final single object or a final (sub)clause and utterances containing a nonfinal object being the first item of an enumeration or an initial (sub)clause (see Table 2). To this end, pauses at the end of the selected sample parts were removed, entailing that the boundary marker “pause” was lost during perceptual evaluation, and only pitch movements and preboundary lengthening could be taken into account by the listeners in making their judgements.

In the Emotional Prosody samples, the introductory phrase was removed (see Table 2 for more details). This yielded the Dutch equivalent for the sentence “It's almost time,” uttered in four different ways: neutral, angry, happy, or sad.

### 2.4. Listeners

Three judges, all speech-language pathologists well experienced with dysarthric speech, rated the samples perceptually. The rating took place in a quiet office, with the judges listening to the stimuli and independently noting down their responses on a scoring sheet. Samples were presented only once, using Creative SBS260 speakers connected to a Dell Vostro computer.

Before the actual rating of a prosodic function took place, the judges received detailed instructions concerning the rating system and then rated some practice samples to get accustomed to the rating goals.

Within each prosodic function block, samples were presented in a randomised order with respect to speaker group (PD or CS) and elicitation method (reading or imitation). Only the Focus samples were further subdivided and presented per prosodic pair for the sake of simplicity during the rating.

For the Boundary Marking task, judges had to indicate whether they considered an utterance finished or not finished. In case of doubt, they could tick the option “I don't know.” As far as the Focus task was concerned, judges had to indicate which one of two possible target words they considered to be emphasised. They could also indicate nothing if they did not hear a word being stressed. The Sentence Typing task required the judges to identify utterances as questions or statements. They could also indicate the option “I don't know.” During the Emotional Prosody task, judges had to decide whether an utterance sounded angry, happy, sad, or emotionally neutral. They could also choose the option “unspecified emotion.”

### 2.5. Preparation of the Data for Statistical Analysis

Judges' identification scores were conflated by applying majority rule: if at least two out of three judges had assigned a particular score, that score was retained as the majority score. If this majority score agreed with the prosodic target, a sample was scored as “correctly identified,” if not, it was scored as “not correctly identified.” When all judges had a divergent opinion, a sample was immediately scored as “not correctly identified.”

### 2.6. Ethical Committee

All PD participants signed an informed consent, approved by the Ethical Committee of the Antwerp University Hospital.

## 3. Results

Each of the four tasks was analysed separately using the chi-square test (IBM SPSS version 19 and SAS version 9.2), to examine the influence on listener identification scores (ID-scores) of the independent factors speaker group (control subjects versus PD patients) and elicitation method (reading versus imitating).  Table 4 summarises the results for all functions per speaker group (CS versus PD).  Table 5 presents an additional analysis per severity group, by splitting the PD group into a PD1 group (*n* = 11) with mild dysarthria and a PD2 group (*n* = 7) with moderate or severe dysarthria.  Table 6 lists the results for comparison of elicitation methods within speaker groups.

Considering the percentage of correct ID-scores in general, it can be stated that results for both CS and PD speaker groups are relatively high for most functions (range 78.6%–98.5%), except for Emotion (range 47.7%–63.6%).

### 3.1. Effect of Speaker Group on Correct Identification Scores

Table 4 shows the percentage of correct identification scores by speaker group. The CS group had significantly higher correct ID-scores than the PD group for three cases: imitation of Boundary Marking (*P* = 0.022), imitation of Focus (*P* = 0.038), and imitation of Sentence Typing (*P* = 0.011).

A similar pattern emerges from the analysis in Table 5, taking into account severity rate. A significant difference between groups could be demonstrated in the same three cases: imitation of Boundary Marking (*P* = 0.004), imitation of Focus (*P* < 0.001), and imitation of Sentence Typing (*P* = 0.004). A post hoc pairwise comparison of groups, using Bonferroni correction, showed that the CS group received significantly higher ID-scores than the PD2 group for imitation of Boundary Marking (*P* = 0.009), imitation of Focus (*P* = 0.003), and imitation of Sentence Typing (*P* = 0.006). The PD1 group also had significantly higher ID-scores than the PD2 group for imitation of Focus (*P* = 0.006).

Comparison of the speaker groups yielded no significant differences as far as reading or imitating Emotional Prosody task was concerned.

### 3.2. Effect of Elicitation Method on Correct Identification Scores

Table 6 shows the correct ID-scores for reading and imitation, by speaker group. For the CS and PD1 groups, no significant differences were found between reading tasks and imitation tasks. For the PD2 group, for Focus, there was a significantly lower score (*P* = 0.033) on imitating (81.0%) than on reading (97.2%).

### 3.3. Interrater Agreement

An interrater reliability analysis was performed using the Fleiss kappa statistic (included in freeware “R”, package “irr”) to determine consistency among the three raters. Results for interrater agreement are summarised in Table 7. For Boundary Marking, Focus, and Sentence Typing, agreement was good (*K* > 0.75). For Emotion, on the other hand, the agreement was moderate. 

## 4. Discussion

The answer to research question (1) is positive for three prosodic functions: professional listeners have more difficulty in correctly identifying Boundary Marking, Focus, and Sentence Typing produced by PD speakers compared to healthy speakers. Research question (2a) is answered by the finding that these observed differences only emerge during imitation tasks, not during reading tasks. The answer to research question (2b), on the other hand, is that within groups reading or imitation performances do not differ significantly, with one exception. A more detailed discussion of these findings and their relation to previous research is given below.

The CS group performed significantly better than the PD group and especially the PD2 group on *Boundary Marking* during imitation, but no significant differences occurred during reading. Interestingly, MacPherson and colleagues [14] did demonstrate an impaired differentiation of final and nonfinal syntactic boundaries in PD individuals reading a passage containing subclauses and lists. An intonational analysis revealed a decreased use of falling contours in final boundaries and an increased use of falling contours in nonfinal boundaries. These different results can possibly be explained by the fact that the study focussed on F0 measures, while the present study allowed listeners to make their judgements on the broader basis of all possible prosodic aspects involved, except pause duration at major boundaries. Presumably, PD and CS group performances in this study are comparable, either because both groups produced Boundary Marking in a very similar way or because our PD patients successfully traded prosodic cues in order to obtain a similar result.

CS and PD speakers performed equally well on *Focus* tasks during reading, but CS speakers did better on imitation of Focus tasks, compared to PD and PD2 speakers. This is at odds with a study by Pell and colleagues [5], who required participants to answer a question correctly by reading a printed sentence and producing contrastive stress on one of three possible keywords. Listeners experienced greater trouble in correctly identifying the emphasised words in PD individuals, compared to healthy speakers. Possibly, the presence of visual cues (target words underlined and in bold) in our Focus reading task could partly explain why PD participants in this study managed to perform so well on this specific task, regardless of their severity rate. On the other hand, our findings are in line with previous research on contrastive stress in patients with dysarthria due to cerebral palsy [4], where listeners were also highly accurate at identifying intended stress locations in normal as well as dysarthric individuals. It was argued that the dysarthric speakers relied more heavily on duration and in this way compensated for any losses in range and flexibility in F0 and intensity. We conjecture that such cue trading mechanisms may also help to explain the very high scores of our PD and especially PD2 patients during reading Focus tasks. If, for example, F0, a key acoustic cue for Focus, is difficult to control for PD2 patients, intensity and/or duration cues can be exploited to compensate for F0.

As far as *Sentence Typing* is concerned, PD2 speakers were markedly less successful than healthy or PD1 speakers at conveying the difference between a question and a statement through imitation but performed not significantly worse when reading. Earlier research on the question-statement contrast [5, 20] showed that listeners are quite able to identify statements produced by PD speakers but have far more trouble in correctly identifying questions. Further analysis is required to see if the same phenomenon applies to the current study.

For the *Emotional Prosody* task, no significant differences could be found between groups. This does not tally with findings by Pell and colleagues [5], who were able to demonstrate that PD speakers are generally less successful than healthy individuals at conveying emotions (in particular anger and disgust) during reading. It also does not seem to fit in with an acoustic study by Möbes and colleagues [13], who discovered that PD patients, compared to healthy speakers, have a significantly smaller F0 and intensity range when pronouncing a name with a particular emotion, but similar ranges during imitating.

The Emotional Prosody tasks in the present study clearly generated very low percentages of correct ID-scores compared to the other three functions. Admittedly, rendering a posed emotion on command is not an easy task for the speaker, but there are also a few aspects which presumably aggravated the task for the listener as well. First, judges had to take a dichotomous decision in the case of Boundary Marking (utterance finished or unfinished?), Focus (accent on word x or y?), and Sentence Typing (question or statement?), whereas in the case of Emotional Prosody, they had to choose between five options (neutral, angry, happy, sad, and unspecified emotion). Second, some of the target emotions are acoustically quite related: angry and happy speech, for example, are both characterised by increased levels of pitch, intensity, and speech rate [28, 29] and show a very similar pattern in terms of spectral properties [30]. Third, listeners may have mutually divergent internal representations of the specific emotions. All these factors might also help to explain why interrater agreement for Emotion is fairly low, as opposed to the other prosodic functions, for which interrater agreement was considered good.

Summarising, it can be stated that PD speakers proved to be capable of conveying Boundary Marking, Focus, and Sentence Typing through reading tasks in a successful manner, not significantly different from healthy speakers. This result was counterintuitive to the first author's occasional initial impression of PD participants' poor prosodic abilities before systematically assessing them. Possibly, the assessment setting stimulated PD speakers to focus their attention on making good use of their preserved prosodic abilities. Husbands or wives who had the opportunity of observing the assessment afterwards frequently remarked that their PD partner talked more intelligibly, with a better speaking rate, more volume, and/or more intonation during the assessment compared to everyday life conversation. Further research concerning the possible role of cognitive factors such as attention is necessary to reveal whether the stated claim can be substantiated. In addition, an acoustic analysis of the reading tasks is needed to delineate the exact share of the cue trading mechanism in the success rate obtained by PD speakers.

The question remains why PD speakers, compared to healthy speakers, perform worse during imitation. It needs to be recognised that imitation involves both perception and production. Possibly, PD speakers have a harder time perceiving the prosodic characteristics they have to imitate and are therefore less capable of imitation or adaption of their speech to a model, even more significantly so in the case of a more severe dysarthria. The fact that reading tasks yielded no significant differences between CS and PD speakers seems to point in this direction as well. It has already been shown that individuals with PD are less able to discriminate prosodic forms (see review by Schröder et al. [19]) and are less sensitive to prosodic meanings during speech comprehension tasks (see discussion and references in [5]). This leads us to the conclusion that the assessment of perception and comprehension of prosody in PD speakers will have to be addressed separately, in order to enable clinicians to localise the prosodic problem more accurately.

As far as the influence of elicitation method within speaker groups is concerned, all speaker groups generally perform alike on reading tasks compared to imitation tasks, with one notable exception. Apparently, PD2 speakers are perfectly capable of realising Focus when reading but have trouble imitating Focus utterances correctly. Focus allows for quite some cue trading in reading tasks (see Table 1), but the imitation task forced the PD2 speakers to copy the model as faithfully as possible, relying mainly on F0 to render Focus. Having to give up on cue trading might have caused the worse results for the Focus imitation task.

A final remark and possible limitation of the current study concerns the fixed order in which tasks were administered: all participants first undertook the reading tasks and then the imitation tasks. The rationale behind this practice was to avoid any learning effect during the assessment: when a speaker hears model utterances during imitation tasks first, this could possibly influence and shape his performance during the reading tasks later on.

## 5. Conclusions and Future Directions

Professional listeners experienced greater trouble in correctly identifying Boundary Marking, Focus, and Sentence Typing in imitation tasks produced by PD speakers (particularly subjects with a moderate or severe dysarthria), compared to healthy speakers. Emotional Prosody proved to be a task with very low correct identification scores for all speaker groups, with no demonstrable between-group differences. Low interrater agreement presumably reflects the difficulty of this task for the judges. Comparison of the effect of elicitation methods revealed no big differences in performance within speaker groups, except in the case of moderately or severely dysarthric individuals conveying Focus in a much less effective way during imitation than during reading. These findings, resulting from the assessment in its current format, raise some issues we would like to address as we move forward.

Firstly, healthy individuals performed systematically better than PD patients (especially with a moderate or severe dysarthria) on imitation compared to reading, except in the case of Emotion. This gave rise to the hypothesis that perhaps PD patients have problems in adequately perceiving and/or comprehending prosody in the model utterances. In order to gain further insight in this matter, an assessment method for the perception and comprehension of prosody in dysarthric patients will be developed and tested on dysarthric PD patients in the near future.

Secondly, the deviant results for Emotional Prosody compel us to rethink the perceptual judgement procedure. A new line of thought could be to judge emotions along a broader dimension of arousal and classify utterances as active or passive [28, 29]. Next to that, a break-down analysis per emotion will be carried out to find out possible differences between emotions.

Thirdly, both (acoustic) cue trading and visual (typographical) cues might have possibly helped PD patients to perform well on the Boundary Marking, Focus, and Sentence Typing reading tasks. An acoustic analysis will have to clarify the role of the cue trading mechanism throughout the various prosodic functions. Also, the effect of cues on PD patients' prosodic communicative efficiency needs further attention.

Fourthly, results pertaining to the effect of severity rate were obtained on the basis of rather small and uneven PD speaker groups. Analysis of an enlarged and more balanced dataset with respect to severity will have to clarify whether differences could be found between control speakers and PD speakers with a mild dysarthria and whether more differences could be found between speakers with a mild versus moderate or severe dysarthria. When collecting new PD samples, clinical patient data will be gathered as well.

Finally, remediation of prosodic communicative functions, currently still unreclaimed territory, also deserves further attention and research. Therefore, a treatment programme paralleling the assessment battery will be designed. Professional listeners' judgements will be used as a basis to develop an automated assessment and treatment programme.

## Figures and Tables

**Table 1:** Prosodic functions in Dutch: classification and perceptual correlates based upon Rietveld and van Heuven [23].

Name	Prosodic Function	Perceptual correlates (for normal speech)	Name in current assessment
Lexical function	Discriminates between words	**F0 change** (vowel) duration intensity	Lexical Stress

Phrasing function	Segments the speech stream in information units	**Preboundary lengthening** pauses F0 change	Boundary Marking

Attentional marking	Highlights the most important elements in a unit	**F0 change** (vowel) duration intensity	Focus

Intentional marking	Nuances meaning	**F0 change**	—

Sentence typing	Discriminates between questions (Q) and declarations	**Final F0 rise (Q)** General F0 rise (Q) High initial F0 (Q)	Sentence Typing

Emotional prosody	Discriminates between different emotional states	**General F0 **	Emotional Prosody
		**F0 span**	
		**speech rate**	

Perceptually most prominent correlates according to [23] are printed in bold.

**Table 2:** Overview of the reading and imitation tasks; all task items included (English translations in italics).

Boundary Marking task	
(1a) Hij kocht een jas, [een broek en een trui.]	(1b) Hij kocht een jas.
*He bought a coat, [trousers, and a sweater.]*	*He bought a coat.*
(2a) Geen nieuws, [goed nieuws.]	(2b) [Op dit moment is er] geen nieuws.
*No news [is good news.]*	*[At this moment there is] no news.*
(3a) Als ik ziek ben, [blijf ik thuis.]	(3b) [Ik blijf thuis,] als ik ziek ben.
*When I'm ill, [I stay at home.]*	*[I stay at home,] when I'm ill.*

Focus task	

(1a) Ze **wil** geen telefoon meer krijgen.	(1b) Ze wil geen **telefoon** meer krijgen.
*She doesn't **want** to get any more calls.*	*She doesn't want to get any more **calls**.*
(2a) **Luc** werkt in het ziekenhuis.	(2b) Luc werkt in het **ziekenhuis**.
***Luke** works at the hospital.*	*Luke works at the **hospital**.*
(3a) Misschien heeft **Piet** vakantie.	(3b) Misschien heeft Piet **vakantie**.
*Maybe **Pete**'s on holiday.*	*Maybe Pete's on **holiday**.*

Sentence Typing task	

(1a) Karen speelt tennis.	(1b) Karen speelt tennis?
*Karen plays tennis.*	*Karen plays tennis?*
(2a) Je hebt de lotto gewonnen.	(2b) Je hebt de lotto gewonnen?
*You've won the lottery.*	*You've won the lottery?*
(3a) Hij kocht een jas.	(3b) Hij kocht een jas?
*He bought a coat.*	*He bought a coat?*

Emotional Prosody task	

(1a) Het is bijna tijd.	(1b) [Schiet op!] Het is bijna tijd!
*It's almost time.*	*[Hurry up!]* *It's almost time! *
	(2b) [Fantastisch!] Het is bijna tijd!
	*[Fantastic!] It's almost time!*
	(3b) [Spijtig.] Het is bijna tijd.
	*[Too bad.] It's almost time.*

Sample parts removed to obtain prosodic minimal pairs for scoring are between [].

Words to be stressed in the Focus task are underlined and in bold print.

**Table 3:** PD speaker characteristics.

Speaker	Gender	Age (years)	Severity of dysarthria	After onset of dysarthria (years)
PD01	F	74	2	Unknown
PD02	M	75	1	3
PD03	M	82	3	Unknown
PD04	M	63	1	2
PD05	M	70	1	1
PD06	F	63	1	2
PD07	F	47	1	4
PD08	F	79	2	5
PD09	M	52	1	5
PD10	F	61	1	3
PD11	F	47	1	3
PD12	F	55	1	1
PD13	F	73	1	2
PD14	F	58	2	Unknown
PD15	M	52	2	5
PD16	F	60	2	1
PD17	F	65	2	2
PD18	M	64	1	4

PD: subject with Parkinson's disease; M: male; F: female; severity of dysarthria scale: 1: mild; 2: moderate; 3: severe.

**Table 4:** Results for 2 speaker groups: % correct identification scores and *P* values resulting from  **χ*²* test (statistically significant *P*  values in bold print).

Task	Elicitation method	% correct ID-score		Significance level
		CS (*n* = 18)	PD (*n* = 18)	*P* value
Boundary Marking	Reading	92.6	90.2	0.535
	Imitation	97.1	88.9	**0.022**

Focus	Reading	94.4	97.1	0.500*
	Imitation	98.0	91.7	**0.038**

Sentence Typing	Reading	91.7	85.3	0.147
	Imitation	96.1	86.1	**0.011**

Emotional Prosody	Reading	61.1	48.5	0.135
	Imitation	63.2	61.1	0.796

CS: control speaker group; PD: Parkinson's disease speaker group; *: Cases where conditions for a valid  **χ*²* test were not met and Fisher's exact test was used instead.

**Table 5:** Results for 3 speaker groups: % correct identification scores and *P*-values resulting from **χ*²* test (statistically significant *P*-values in bold print).

Task	Elicitation method	% correct ID-score			Significance level
		CS (*n* = 18)	PD1 (*n* = 11)	PD2 (*n* = 7)	*P* value
Boundary Marking	Reading	92.6	92.4	86.1	0.456
	Imitation	97.1	93.1	80.6	**0.004**

Focus	Reading	94.4	97.0	97.2	0.645
	Imitation	98.0	98.5	81.0	<0.001*

Sentence Typing	Reading	91.7	87.9	80.6	0.188
	Imitation	96.1	90.9	78.6	**0.004**

Emotional Prosody	Reading	61.1	47.7	50.0	0.322
	Imitation	63.2	63.6	57.1	0.830

CS: control speaker group; PD1: Parkinson's disease speaker group with mild dysarthria; PD2: Parkinson's disease speaker group with moderate or severe dysarthria; *: Cases where conditions for a valid  **χ*²* test were not met and Fisher's exact test was used instead.

**Table 6:** Results for elicitation method: % correct identification scores and *P* values resulting from  **χ*²* test (statistically significant *P* values in bold print).

Task	Speaker group	% correct ID-score		Significance level
		Reading	Imitation	*P* value
Boundary Marking	CS	92.6	97.1	0.147
	PD	90.2	88.9	0.757
	PD1	92.4	93.1	1.000*
	PD2	86.1	80.6	0.527

Focus	CS	94.4	98.0	0.281*
	PD	97.1	91.7	0.092
	PD1	97.0	98.5	1.000*
	PD2	97.2	81.0	0.033*

Sentence Typing	CS	91.7	96.1	0.179
	PD	85.3	86.1	0.866
	PD1	87.9	90.9	0.572
	PD2	80.6	78.6	0.829

Emotional Prosody	CS	61.1	63.2	0.796
	PD	48.5	61.1	0.135
	PD1	47.7	63.6	0.133
	PD2	50.0	57.1	0.606

CS: control speaker group; PD: entire Parkinson's disease speaker group; PD1: Parkinson's disease speaker group with mild dysarthria; PD2: Parkinson's disease speaker group with moderate or severe dysarthria; *: Cases where conditions for a valid  **χ*²* test were not met and Fisher's exact test was used instead.

**Table 7:** Interrater agreement results, obtained through a Fleiss Kappa statistic.

Task	Fleiss Kappa coefficient
Boundary Marking	0.7626
Focus	0.8889
Sentence Typing	0.8327
Emotional Prosody	0.4430
